# Clitoral keloids after female genital mutilation/cutting

**DOI:** 10.4274/tjod.32067

**Published:** 2016-09-15

**Authors:** Özer Birge, Murat Akbaş, Ertuğrul Gazi Özbey, Mehmet Adıyeke

**Affiliations:** 1 Nyala Sudan Turkey Training and Research Hospital, Clinic of Gynecology and Obstetrics, Nyala, Sudan; 2 Okmeydanı Training and Research Hospital, Clinic of Obstetrics and Gynecology, İstanbul, Turkey; 3 Nyala Sudan Turkey Training and Research Hospital, Clinic of Urology, Nyala, Sudan; 4 Bergama State Hospital, Clinic of Gynecology and Obstetrics, İzmir, Turkey

**Keywords:** Female genital mutilation, infibulation, circumcision, keloids, vulvar mass

## Abstract

We aimed to describe the presentation of long-term complications of female genital mutilation/cutting and the surgical management of clitoral keloids secondary to female genital mutilation/cutting. Twenty-seven women who underwent surgery because of clitoral keloid between May 2014 and September 2015 in Sudan Nyala Turkish Hospital were evaluated in this retrospective descriptive case series study. The prevalence of type 1, type 2, and type 3 female genital mutilation/cutting were 3.7%, 22.2%, and 74.1%, respectively (type 1: 1/27, type 2: 6/27, and type 3: 20/27). All patients had long-term health problems (dysuria, chronic pelvic pain, vaginal discharge, and chronic pruritus) and sexual dysfunction. Keloids were removed by surgical excision. There were no postoperative complications in any patient. Although clitoral keloid lesions can be seen after any type of female genital mutilation/cutting, they usually develop after type 3 female genital mutilation/cutting. Most of these keloids were noticed after menarche. Keloids can be removed by surgical excision and this procedure can alleviate some long-term morbidities of female genital mutilation/cutting.

## INTRODUCTION

Female genital mutilation/cutting (FGM/C) is the medically unnecessary modification of the female external genitalia for cultural reasons, which leads to dysfunction^(1)^. Historically, there have been references to its existence in Ancient Egypt; no one actually knows when, how or why FGM/C began. It is important to note that there have been no medically documented justifications that show the benefits of this practice for the purpose of enhancing woman’s health. The procedure is named “female circumcision” in countries where it is practiced, but the term “FGM” is used in medical literature because of its harmful physical and psychological consequences^(2)^. It is often performed before adolescence. FGM/C is still known to be practiced in approximately 30 countries in Africa, in a few countries on the Arab Peninsula, among some communities in Asia, and among immigrants from these areas who have settled in Europe, Australia, and North America^(2)^. It affects approximately 100 million women worldwide and another 2 million procedures are performed each year^(2,3)^.

Although the practice may show variations from one country to another, it is performed secretly because it is an illegal practice. Typically, the procedure is undertaken by a traditional circumciser using a sharp blade or razor, which is not sterilized, and without any anesthesia^(4,5,6)^.

The World Health Organization (WHO) has categorized FGM/C into four main groups:

**Type 1:** Amputation of the prepuce, sometimes along with partial or total removal of the clitoris (sunna).

**Type 2:** Amputation of the clitoris and part or all of the labia minora (excision).

**Type 3:** Amputation of the clitoris and labium minora. Cutting of the labium majora and sutured wound edges. A small opening is created to allow the flow of urine and menstrual blood (infibulation).

**Type 4:** A new category that encompasses a group of other operations on the external genitalia including piercing or incising the clitoris and/or labia, stretching the clitoris and/or labia, cauterization, scraping and/or cutting of the vagina, introduction of corrosive substances and herbs into the vagina, and similar practices^(7)^.

Pain, hemorrhage, and infections are the three most important early complications. The long-term complications, especially as related to type 3 (infibulation) and these are infertility, vulvar mass-keloids, vesicovaginal fistula, and vesicourethral fistula, menstrual irregularities, chronic cystitis and dysuria, chronic pelvic pain, and dyspareunia. Maternal and fetal mortality and morbidity is also increased due to dystocia^(8,9)^. A number of studies also concluded that FGM/C had adverse effects on circumcised women’s sexual life, leaving them feeling inadequate at intercourse^(10,11)^. This study describes the presentation of long- term complications of FGM/C and the surgical management of clitoral keloids.

## CASE REPORT

This was a retrospective study of the case notes on 27 patients with FGM/C who had clitoral masses and were referred to Sudan Nyala Turkish Hospital between May 2014 and September 2015. Gynecologic history, long-term complications of FGM/C, size of masses, and short-term complications after surgery were recorded. Surgical excision was performed for all patients by the same surgeon and all specimens were evaluated by the same pathologist.

Twenty-seven patients with clitoral mass were admitted to our gynecology outpatient clinic. All of the patients had a history of undergoing FGM/C. All masses were pre-diagnosed as clitoral keloid before surgery. The demographics and gynecologic history of the patients are shown in Table 1. The mean age of the patients were 18.07±7.16 years. FGM/C had been performed on all patients when they were aged between 7 to 9 years. The mean time between the procedure and notification of vulvar mass was 4.37±1.96 years. The sizes of the masses varied between 3 to 10 centimeters. The mean time between notification of the mass and referral to a gynecologist was 6.15±5.76 years.

The percentages of FGM/C types and symptoms are shown in Table 2. Most of the patients had type 3 FGM/C (n=20), which is the most destructive procedure and has the worst prognosis. The most common symptoms were dysuria with chronic cystitis and dyspareunia. All patients had at least two long-term health problems.

**Figure 1:**
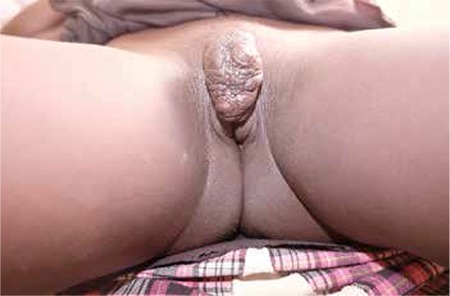
Vulvar mass before surgery

**Figure 2:**
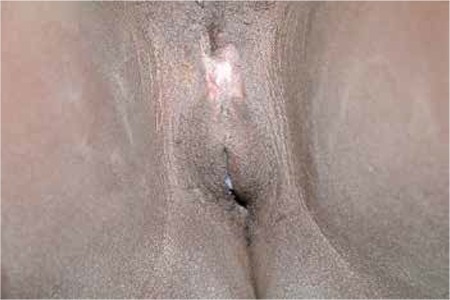
After surgery

Surgical excision was performed for all patients. There were no early complications recorded in any of the patients. Pathologically all masses were reported as keloid. Pictures of one patient before and after the surgery are shown in Figures 1 and 2.

## DISCUSSION

Currently, over 100 million women throughout the world have been subjected to the practice of FGM/C. Likewise, 66.000 women in the United Kingdom and 50.000 women in France have been reported^(12)^. The age at which girls undergo FGM/C is mostly before 12 years^(1,2,7)^. In our study, all patients underwent FGM/C between the ages of 7 and 9 years.

FGM/C is accepted as an assault on the human rights of women by the WHO because the practice deprives women of their rights to experience their sexuality. Its detrimental psychological and psychosexual lifelong effects on women’s sexual life have been examined in many studies. The psychotherapist and social activist Leila Hussein’s case can be given to show the seriousness of this non-medical practice. In her report to the Guardian, she stated that she recalled every single detail: She was cut when she was seven years old, four women held her down, she felt every single cut, and she screamed so much that she fainted^(13)^. Girls who have not been circumcised are considered sexually active and labeled as “ghalfa,” which is used for a woman who is sexually free and not respectful, who has the potential not to show fidelity to her family; as such these girls would be a target for abuse in their schools and social environments^(12)^. In order to protect their daughters from this kind of abuse, families choose to have their daughters circumcised for concerns of virginity when their daughters marry.

A significant number of children undergo FGM/C when under 1 year of age, which concurs with the global trend of FGM/C occurring at an increasingly younger age. This reduces the chance of the child remembering or being aware that the practice has taken place, thus reducing the chances of presenting to a physician^(14)^. In our study, all patients were aged between 7 to 9 years when FGM/C was performed.

The dermatologic findings of FGM/C have been extensively reported in case reports and include keloids, epidermoid cysts, clitoral neuromas, and scarification. Women may delay treatment of keloids in the genital region for years because of embarrassment or fear of surgical options. Large keloids can contribute to obstetric complications^(15)^. We found that the mean time between notification of keloid and referral to a gynecologist was 6.15±5.76 years; this delay was more than ten years in six patients.

Allah et al.^(16)^ performed surgical excision for 149 patients with keloids. The recurrence rate was 100%. The authors concluded that keloids were not homogeneous biologic entities and were related with increased immunologic factors. The best prevention is to avoid the scar itself^(16^). Gurunluoglu et al.^(17)^ reported a case of clitoral keloid that developed after a traumatic laceration. The keloid was treated with surgical excision, followed by silastic sheet application for six months. The sizes of keloid lesions were between 3 to 10 centimeters in our study. We performed surgical excision for all patients. There were no early complications recorded in any patients, but we did not have results for long-term follow-up.

Women with FGM/C have a significantly higher prevalence of long-term health problems such as dysmenorrhea, vulvar or vaginal pain, problems related to anomalous healing (e.g., fibrosis, keloid, synechia), and sexual dysfunction. They are also much more likely to suffer complications during delivery (perineal tear, obstructed labor, episiotomy, cesarean, stillbirth), and complications associated with anomalous healing. Similarly, newborns were found more likely to suffer complications such as fetal distress and caput of the fetal head^(18)^. In our study, the patients’ most common symptoms were dysuria, dyspareunia, and pruritus. All case notes were obtained from the gynecology clinic; therefore, we had no data about obstetric complications.

Despite prohibition, FGM/C is performed on girls illegally. These women have significantly higher prevalence of long-term health problems related to the genitourinary system. In cases of vulvar mass, keloid development secondary to FGM/C should be considered for immigrant patients. Keloids can be removed by surgical excision and this procedure can alleviate some long-term morbidities of FGM/C.

## Figures and Tables

**Table 1:**
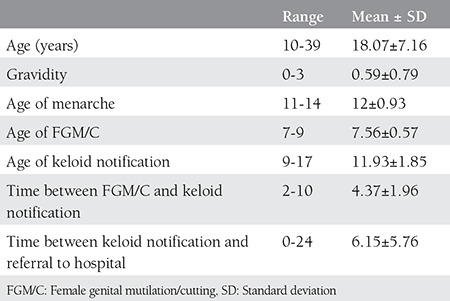
The demographics and gynecologic history of the patients

**Table 2:**
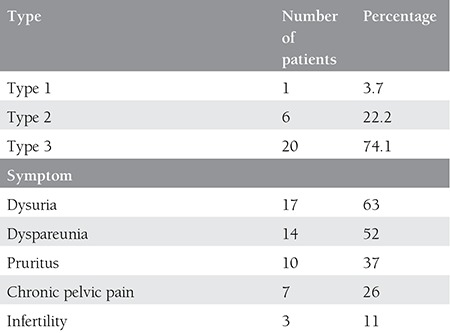
Percentages and numbers of female genital mutilation/cutting types and symptoms
